# Immune Response in Ovarian Cancer: How Is the Immune System Involved in Prognosis and Therapy: Potential for Treatment Utilization

**DOI:** 10.1155/2010/791603

**Published:** 2011-01-24

**Authors:** Nikos G. Gavalas, Alexandra Karadimou, Meletios A. Dimopoulos, Aristotelis Bamias

**Affiliations:** Department of Clinical Therapeutics, Medical School, University of Athens, Alexandra Hospital, 80 Vasilissis Sofias Avenue, 115 28 Athens, Greece

## Abstract

Ovarian cancer is one of the leading causes of cancer-related death among women. Resistance to the disease occurs in more than 70% of the cases even after treated with chemotherapy agents such as paclitaxel- and platinum-based agents. The immune system is increasingly becoming a target for intense research in order to study the host's immune response against ovarian cancer. T cell populations, including NK T cells and Tregs, and cytokines have been associated with disease outcome, indicating their increasing clinical significance, having been associated with prognosis and as markers of disease progress, respectively. Harnessing the immune system capacity in order to induce antitumor response remains a major challenge. This paper examines the recent developments in our understanding of the mechanisms of development of the immune response in ovarian cancer as well as its prognostic significance and the existing experience in clinical studies.

## 1. Introduction

Cancer is one of the leading causes of death in the developed world outnumbering even heart disease in the United States [1]. In turn, ovarian cancer remains the leading cause of death among gynaecological malignancies and is the fourth most common cause of cancer-related death among women. Epithelial ovarian cancer is the main type of the disease accounting for more than 90% of all malignant ovarian tumors. According to the initial FIGO stage, the prognosis of ovarian cancer varies; a 5-year survival reaches 90% when the disease is confined within the ovary, but it drops to below 50% for the cases that cancer has spread outside the pelvis. Ovarian cancer is usually diagnosed in advanced stages (FIGO stages III and IV), and prognosis is generally rather poor. Major established prognostic factors, apart from FIGO stage of the disease, include tumor grade, histologic subtype, and the volume of disease remaining after cytoreductive surgery [2]. Nevertheless, the value of these factors in a population with advanced stage and usually high-grade tumors is limited.

Current treatment of advanced ovarian carcinoma includes debulking and chemotherapy, mainly the combination of the use of paclitaxel and platinum agents and at least 70% of the patients treated with the above combination initially respond to treatment. Intraperitoneal drug administration has substantially improved the survival of patients who have minimal gross disease remaining after surgery and can also tolerate the side effects of aggressive treatment [3]. 

Despite the significant advances in surgery and chemotherapy, the disease is more likely to relapse in about 70% of the cases [4] with resistance being prevalent in most cases. As a result, new ways of treating the disease are currently being explored focusing on the biology of cancer and more specifically within the ovarian tumor microenvironment. Therefore, clinical research has focused on molecular markers, which are related either to the behaviour of the disease or the response to chemotherapy in order to define the outcome in these patients and establish furthermore potential targets for therapy. 

Oncogenesis in all types of cancer, including ovarian cancer, is a process that involves multiple molecular pathways, which regulate important functions of cancer cells. In 2004, the Baltimore group proposed a model for the division of epithelial ovarian tumors into two rather broad categories termed type I and type II, that correspond to two main pathways of tumorigenesis [5].

The major groups are genes involved in apoptosis and cell cycle regulation, genes encoding for growth factors and genes involved in angiogenesis. The prognostic and predictive value of several factors implicated, in these pathways, has been recently studied. Genetic alterations in associated genes, such as mutations of p53, malfunctioning genes of the BRCA family (BRCA1 and BRCA2) in about 15% of inherited types of ovarian cancer [6], malfunction of tumor suppressor genes such as *ARHI* [7], the cyclinE/CDK2 and cyclinD/CDK4 complexes and the cell cycle regulators p27, p15, and p16 have all been studied in this context [8–11]. Although some studies have reported relevant associations, the prognostic role of these factors remains to be elucidated in full. 

**Figure 1:**
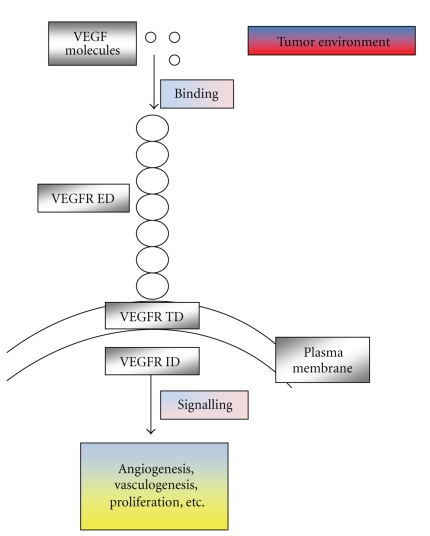
VEGF exerts its signalling effect via its receptor VEGFR. VEGF, mainly the VGEFA isoform exerts its effects via binding its receptor VEGFR (mainly VEGFR2). It is a powerful angiogenic factor that holds a pivotal role in tumor progress and metastasis. It comprises an attractive target for possible agents that will block its function and therefore enhance patients' survival. ID: Intracellular domain, ED: extracellular domain, TD: Transmembrane domain.

Angiogenesis is a critical function for the expansion of a tumor and also for its metastatic potential, and it is influenced by the tumor microenvironment [12]. Its significance in ovarian cancer has been well established, and a number of angiogenic factors have been identified. The vascular endothelial growth factor (VEGF) holds a pivotal role in the angiogenic process [13]. It is produced by cancer cells and assists tumor progression and metastasis (Figure 1) exerting a central role in the formation of ascitic fluid and metastasis in the peritoneum. It is also related to the invasive and metastatic potential of ovarian cancer [14–16]. 

Immune surveillance has long been recognized as an important element of host anticancer response. Agents which augment immune response as well as antibodies against certain tumor antigens have been approved for the treatment of different types of neoplasms. In the recent years, we have witnessed important developments in our understanding of cancer immunology. Many of these developments involve ovarian cancer, and this paper will focus on them.

## 2. Cancer and the Immune System

The immune system responds to the presence of cancer antigens. A key advance in recent advances in immunology has been the elucidation of antigen-specific cell recognition and destruction of target cells. Mutations can occur in common antigens that are found in otherwise normal functioning genes in the cell; these were initially termed the tumor-specific antigens [17], and on those that can be found in both normal and cancer cells called the tumor-associated antigens (TAA) [18]. This terminology is still extensively used but it has been termed as imperfect by researchers and although still present in the literature, other modern antigen classifications have emerged based on the antigens' molecular structure and source. More modern terminology divides antigens into categories such as differentiation antigens and overexpression antigens [19] and also viral antigens. A distinct example of the latter category is Epstein-Barr Virus Nuclear Antigen (EBNA-1), which is associated with Burkitt's lymphoma and nasopharyngeal carcinoma [20]. Identifying tumor antigens has been an ongoing process with a number of techniques, having been employed, based on several components of the immune system [21–24]. 

In contrast to early theories that a tumor could not elicit an immune reaction, later experiments showed that it actually does provoke the onset of an immune response [25–27]. More specific studies have shown that both the innate and adaptive “arms” of the immune system are implicated in antitumor response [28, 29]. There is a number of components of the immune system that have been implicated with cancer cell elimination, equilibrium, and also escape from immune surveillance; all three comprising what is called “immunoediting” [30], a process that emphasizes in the dynamic interaction of the immune system with cancer, and it is present in almost all types of tumor including ovarian cancer. It is a process that has been reinforced in the last few years for its usage in cancer progress. Immunoediting is divided in elimination, equilibrium, and escape. At first, cancer is eliminated, rendered nondetectable, followed by a period of being kept in check by the immune system, and finally cancer becomes clinically detectable when it has escaped antitumor immunity. Thus the immune system protects the host from cancer and it also plays a role in “sculpting” immunogenicity, and this has actually been experimentally shown [31]. 

Elimination and equilibrium are achieved via lymphocytes, mainly the T cell subpopulation [32]. In cancer patients the “healthy” response against the tumor is counteracted by a suppressive, tumor-driven effect. This hypothesis is strengthened by recent studies showing that the absence or presence of T cells in colorectal cancer specimens more accurately predicted the outcome than using standard prognostic factors [33]. Other studies in different types of tumor, mainly cervical and breast cancer, have also shown similar results [34, 35]. These studies further confirmed the importance of the immune response in prognosis alongside other more established factors. Recent studies also support the case of immunoediting by observing that tumor infiltration by lymphocytes is linked to tumor-associated immune response, mainly showing that the presence of tumor infiltrating lymphocytes may be associated with improved prognosis and clinical outcome in cancer patients [36–38] including ovarian carcinoma [39, 40]. These observations as well as preclinical data also suggest that by enhancing the host immune system, it may achieve tumor destruction and act synergistically with other anticancer therapies.

Although the development of antitumor immune response has been well established, there is also evidence that tumors can escape destruction by suppressing the immune system both within the cancer microenvironment and also on a systemic basis. T regulatory cells (Tregs), for example, that can suppress effector T cells action have been found in the microenvironment of several types of tumor [41–43]. Similar effects on regulation of Tregs can also be brought about in systemic modes of immunosuppression by tumors. For example, an increase in blood Tregs content has been observed in melanoma [44]. In colorectal cancer, increased numbers of activated granulocytes [45] have also been reported. Such cell types have shown to suppress tumor-specific T cells in mouse models [46]. Other types of immunosuppression consist of the downregulation of Major Histocompatibily Complex (MHC) and tumor antigen loss [47]. They also include disruption of specific Natural Killer (NK) cells employment that inhibit immune system-mediated tumor destruction [41, 48].

## 3. Ovarian Cancer and Lymphocyte Response

Epithelial ovarian cancer is characterized by periods of remission and relapse of sequentially shortening duration until chemoresistance occurs [40]. Such patients are the best candidates for immunological studies, since T cells' presence can be utilized as markers for disease progress and can be evaluated at different stages of the disease. The progression of cancer in the peritoneal cavity and the frequent formation of ascites, which characterize advanced stages of ovarian cancer, mainly stage IV, make this tumor a model for the study of different lymphocytic populations. Ascitic fluid as well as peritoneal metastases can be easily obtained through paracentesis, laparoscopy, or open surgery, and cells can be screened by various techniques such as flow cytometry or immunohistochemistry. 

It is believed that the presence or absence of specific populations of T cells, which hold a central role in immunoediting within epithelial ovarian cancer tumors, is associated with important differences in prognosis. Studies in paraffin-embedded tissues have reinforced this notion and have shown that the presence of tumor infiltrating lymphocytes (TIL) such as CD3+ cells and increased number of cytotoxic CD8 lymphocytes were associated with prolongation of survival [49–51]. For example, in the case of CD3 TILs, Tomšová et al. have shown that patients exhibiting higher CD3 cell numbers had an improved overall survival of 60 months over 29 months for patients that had lower CD3 cell numbers. 

Elimination is also conferred by CD3+ CD56+ cells, containing the NK-like T cytotoxic cells which have cytotoxic properties against tumor cells and contain the highest such property among effector killer cells in vitro [52, 53]. Experiments, using blood cells from lymphoma patients, showed significant expansion of this cell population in ex vivo conditions, which accounted for the 20% of a cytokine-induced population that resulted in significant cytotoxicity against cancer cells in vitro [54]. Frozen tissue has also been used in immunohistochemical studies showing similar results [55], where the presence of CD3+ cells was shown in most cancer specimens. In this paper, immunohistochemical studies also showed the presence of CD4+ and CD8+ TILs with numbers that were closely related. Moreover, both types of cells, CD4+ and CD8+, were both present or absent in specimens examined. The 5-year progress-free survival percentage for patients with the presence of TILs according to Zhang et al. was 38%. Nesbeth et al. have recently shown the positive effect of CD4+ T cells in ovarian cancer via the use of a novel mechanism that recruits dendritic cells to the tumor site that in turn activate tumor-specific CD8+ cells which then mediate long-term protection [56].

The presence of CD3+ CD56+ cells in ascitic fluid taken from advanced ovarian cancer patients has been shown to be inversely correlated with the presence of vascular endothelial growth factor (VEGF) [57]. In addition, low CD3+ CD56+ content was correlated with poor prognosis and platinum resistance. NK cells' rapid activation, and cytotoxic activity without need for prior sensitization and the release of cytokines such as IFN-*γ*, TNF-*α*, and IL-10, indicates their importance [58]. Early studies have shown the efficacy of NK cells against tumors when activated by cytokines [59, 60] or when ex vivo stimulated lymphokine-activated killer cells were adoptively transferred into patients [61, 62]. Recent studies though have shown that the expression of mucin (MUC) molecules on the ovarian cancer cell surface, namely, MUC16 which is a carrier for the CA125 tumor marker, assist in the avoidance of the tumor cells' recognition by NK cells [63]. Human Leukocyte Antigen (HLA) class I antigens that can play a negative role in antitumor functionality of NK cells are downregulated in ovarian cancer, hence making the use of NK cells possibly quite important in ovarian carcinoma [64]. This is enhanced by findings that the formation of ascites in late stage ovarian cancer may be inhibited by Cd-1-mediated activation of NK cells [65].

Another factor, termed programmed cell death 1 (PD-L1) which is expressed on tumor cells, has been shown to act as a prognostic factor. Its expression level has been shown to be inversely correlated with CD8+ cell count rendering this protein a factor of poor prognosis, since it has been suggested to directly inhibit CD8+ cells [66].

Dendritic cells migrate in a transendothelial manner via the use of L1 IgCaM molecule as has been recently shown by Maddaluno et al. [67], an observation that may play a role in tumor metastasis. L1 is a glycosylated protein that has been recently reported to be expressed in 40%–70% of cases of epithelial ovarian cancer and is associated with poor prognosis [68].

In contrast to the augmentation of antitumor response by the aforementioned populations, another specific subset of T cells has been shown to play a key role in tumor immunity. T regulatory cells (Tregs) play a key role in peripheral tolerance. Since tumor-associated antigens (TAA) are self antigens, they are subjected to control by peripheral tolerance. Tregs within the CD4+ CD25+ T cell population are characterized by the expression of the FoxP3+ protein [69, 70]. Humans bearing tumors show an elevated amount of Tregs in their blood as well as malignant effusions [71, 72]. Sato et al. [69] identified cells in ovarian tumors expressing both CD25 and FoxP3. Recently, the presence of Tregs in ovarian cancer ascites in comparison to normal ascites has been shown [72]. The presence of Tregs in ovarian tumors has been associated with reduced overall survival [73, 74]. More specifically, Curiel et al. showed for the first time that CD4+ CD25+ FoxP3+ Treg cells correspond to poor clinical outcome in epithelial ovarian cancer. The same study also showed that CD4+ CD25+ CD3+ cell populations were much more concentrated in malignant ascites rather than nonmalignant ones and in blood. It was also shown that CD4+ CD25+ cells were preferentially concentrated in tumor mass rather than in tumor draining lymph nodes. Furthermore, the presence of FoxP3 alone was an independent prognostic factor for progress-free and overall survival.

Therefore, Tregs depletion can be expected to lead to more efficient treatment and better prognosis. Current therapeutic agents may be useful in this respect. Classical cytotoxics, such as cyclophosphamide [75] as well as antibody-based immunotherapy with Trastuzumab have been shown to result in a substantial decrease in the number of Tregs in cancer patients [76]. A recent study has shown selective accumulation of NK-T cells, activated CD4 and CD8 lymphocytes and also Tregs in ascites formed in ovarian cancer [72], which complements previous evidence that tumor-associated lymphocytes are indeed present in ascites [70, 73, 77] and may be important for the immune response against the tumor. These results indicate that the presence of cancer cells can activate lymphocytes and could also result in a parallel accumulation of Tregs that may inhibit CD8-mediated immune response against the tumor as has been suggested before [71, 78]. Recent studies also indicate that in the case of epithelial ovarian cancer, local treatment with interleukin 2 may play a role in converting Tregs into Th17 cells, a new player in the field of cancer immunotherapy, with a concomitant relief of Treg-mediated immune suppression and enhancement of antitumor immunity [79, 80]. Plasmacytoid dendritic cells (PDc) have also been shown to contribute to immunosuppression in ovarian cancer by inducing tumor microenvironment Tregs [81].

Another type of cells of the immune system, namely macrophages, are also found in ovarian cancer [82, 83]. The presence of macrophages in tumors has been associated with tumor growth and metastasis in rodents [84, 85]. Kryczek et al. [83] have shown that the B7-H4^+^ receptor expression, which is a negative T cell regulator on tumor-associated macrophages, in ovarian cancer, induces suppression of T cells encompassing tumor-associated antigens immunity. 

Finally, since the increased concentration of autoantibodies can induce the production of Tregs and clinical studies have reported autoimmune paraneoplastic syndromes (different from autoimmune diseases) [86, 87], there may be links between cancer and autoimmune disease that remain to be elucidated in full. These studies may provide us with a greater insight into Tregs activity and association with ovarian cancer.

Lately, different populations such as vascular lymphocytes have shown the ability to form functional blood vessels, and they may be proven to be an important target for blocking cancer progression [88].

The identification of important subsets of lymphocytes in tumors and ascites from ovarian cancer has led to the study of possible immunomodulatory effects of current therapies. Chemotherapy, in particular paclitaxel, may have a positive effect on the immune response by directly downregulating Tregs [89]. Tregs can also be suppressed by cyclophosphamide as has been exhibited in mouse models [75, 90], and NK cells can be activated at the same time. The use of gemcitabine, which is a nucleoside analog, reduced the number of myeloid suppressor T cells, without reducing cytotoxic cells such as NK cells [91]. Gemcitabine, in association with oxaliplatin and interleukins such as IL-2 and GM-CSF, can have a suppressive effect on Tregs [92, 93]: therefore, it could possibly have a positive effect on reducing drug resistance and influence prognosis and disease outcome.

## 4. Cytokines, Growth Factors and Association with Lymphocytes' Mobility and Response

**Figure 2:**
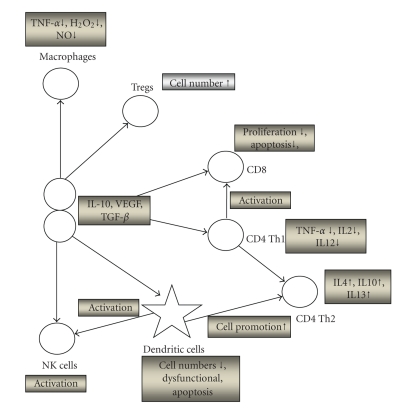
Schematic representation of characteristic immune cells, growth factors, and cytokines interactions in cancer. Interactions between growth factors such as VEGF, cytokines (e.g., TNF*α*) and T cells (e.g., NK, Tregs) are shown in this diagram. Tumor cells bring about the production of cytokines that assist in the mobilization of T cells and induce the production of further cytokines, and they also utilize growth factors such as VEGF to promote neovasculirisation implicated in metastasis. ↑ means increase where ↓ means decrease.

The composition of lymphocytic populations in blood, ascites and tumors is regulated by various cytokines and chemokines produced by the tumors or the components of the immune system. A simple schematic representation of these interactions is depicted in Figure 2.

A number of cytokines have been associated with a direct effect on tumor cells, via surface receptors such as Toll-like receptors [94], but mainly they have been attributed roles in assisting the immune response of the body against tumors. Host antitumor response results from the balance between the T helper 1 (Th1) response, which potentiates immune response and the T helper 2 (Th2) response with a shift in favor of the latter characterising oncogenesis and disease progression. Both Th1 and Th2 immune responses have been associated with the production of cytokines such as Interleukin 12 (IL-12), Interleukin 4 (IL-4), Interferon gamma (IFN-*γ*), Tumor Necrosis Factor (TNF-*α*) (Th1 response), and IL-10 (Th2 response) [16, 95–97]. These cytokines can also be produced by cancer cells; they are present in ascites and have been associated with prognosis in ovarian cancer [71, 98–100]. Gradients between blood and ascites may play a role in migration of leukocytes [101] and factors that facilitate such movements may include L1 [67]. As a consequence, different lymphocytic populations are involved in the two types of response: for example, CD3+ CD56+ cells are associated with Th1 whereas CD4+ CD25+ cells are associated with Th2 response.

The prognostic role of various cytokines has been studied, but no absolutely firm conclusions can be drawn so far. It is conceivable that cytokines involved in Th1 response are expected to predict for better prognosis, while the opposite is expected in those associated with Th2 response. Interleukins in that respect have received much attention. IL-2 initiates the activation of T and NK cells and is also essential for the maintenance of self-tolerance through generation and maintenance of Tregs [102] or by activation-induced cell death [103] to eliminate self reactive T cells. Cytokines such as IL-12 [104] and IL-21 [105] are currently considered for their therapeutic potential in other types of cancer and may have the same effect in ovarian cancer. In glioma, in the case of IL-12, the cytokine is fused with normal glioma cells and dendritic cells and administered to malignant glioma patients [104]. IL-12 is associated with favorable prognosis, and in this study, four patients exhibited a glioma reduction of 50%. For IL-21, Dou et al. have shown that when the gene expressing IL-21 is administered in rodents, it has a positive antitumor effect in squamous cell carcinoma, and therefore IL-21 may be associated with favorable prognosis. This has further been enhanced by a recent study showing that the antitumor effect is increased by human ovarian cancer cells secreting IL21 alone or in combination with GM-CSF [105]. TNF*α* may also be associated with prognosis [72, 106, 107], but reports on whether it is a signature of poor or better prognosis vary. IL-6 levels have been shown to be increased in ovarian cancer patients' serum [108, 109], and it was correlated with poor overall survival. Another cytokine that was shown to be associated with the growth of cancer cells and tumor proliferation is IL-1 [110, 111]. IL-15 has also been recently shown to activate CD8+ and NKT cells that may inhibit tumor growth [112]. Further functional studies are necessary to confirm the above results.

A cytokine that seems to be heavily involved in tumor immunosuppression is transforming growth factor beta (TGF-*β*), a protein that affects proliferation, activation, and differentiation of immune cells and inhibits antitumor immune response [113]. In cancer cells, the production of TGF-*β* is increased, which in turn increases the proteolytic activity of cells and the binding to cell adhesion molecules in the extracellular matrix. TGF-*β* can also convert effector T cells into Tregs [114]. It has been reported that it can also promote angiogenesis and that process can be blocked by anti-TGF-*β* antibodies [115].

TNF*α* is produced by tumor cells and can induce autocrine proliferation and disease progression in ovarian cancer [107, 116, 117]. The autocrine action of TNF*α* may have direct effects on tumor cell spread via acting on the chemokine receptor CXCR4 and also stimulation of blood vessel formation in the peritoneal tumor by inducing expression of VEGF and CXCL12 [118]. In contrast, TNF*α* levels have also been inversely correlated with the presence of CD4+ CD25+ cells, and have been shown to directly downregulate Tregs [119]. This might indicate a favorable effect of this cytokine on prognosis and underlines the complexity of the functions that each of these factors may possess. 

A family of proteins called chemokines (CC) may also be influencing cellular composition in biological fluids. Recent studies have exhibited the detection of mRNA for CCL2, CCL3, CCL4, and CCL5 in solid ovarian tumors by *in situ* hybridization [120]. Moreover, CCL5 has been shown to be secreted by CD4+ T cells, recruits CCR5+ dendritic cells to the tumor location, and activates them through CD40-CD40L interactions [56]. The newly matured dendritic cells prime tumor-specific CD8+ cells thus providing with long term protection. 

In the protein-rich ascitic fluid, different chemokine molecules have been shown to be expressed, with CCL2 being the predominant one [121]. In addition, chemokine stromal-derived factor-1 (CXCL-1) induced the migration of plasmacytoid dendritic cells into the tumor microenvironment in cases of ovarian cancer and induced delivery of survival signals to PDC. In turn, the tumor microenvironmental PDC induced IL-10 expressing Tregs [122], which is correlated to poor prognosis and shorter progress-free survival. Tregs, and IL-10 are associated with poor prognosis in many types of cancer. In the case of Tregs it has been exhibited that CCL22 plays a central role in inducing influx of these cells into tumor sites, and it binds CCR4 that is expressed on Treg surface [123]. 

Interferon gamma (IFN-*γ*) plays a stimulatory role for macrophages turning them from immunosuppressive to immunostimulatory cells [124]. It also skewed monocyte differentiation from tumor-associated macrophages- (TAM-) like cells to M1-polarized immunostimulatory macrophages. Taken together these data show that IFN-*γ* overcomes TAM-induced immunosuppression by preventing TAM generation and functions. 

Furthermore, cytokines such as interleukin 18 (IL-18) [125] and stroma derived factor 1 (SDF-1) [126] have been shown to be correlated with poor prognosis in ovarian cancer patients, but further studies are required to fully evaluate them in the tumor microenvironment and the periphery.

VEGF holds a very important role in the oncogenesis as well as progression and prognosis in ovarian cancer [55, 127]. It is selectively accumulated in ascites and occurs in advanced stages of the disease but not in ascites from cirrhosis [55, 57]. Up to now, this has been attributed solely to its angiogenic properties. Recently, it has been suggested that VEGF also exerts an immunosuppressive effect in cancer, as it was correlated with low levels of IL12, inhibition of dendritic cell maturation, low numbers of NK-T cells, and upregulation of Tregs [58, 59, 128–130]. It can also induce expression of the T cell cosignaling molecule B7-H1 on myeloid dendritic cells (MDC). Barnett et al. [15] have reported that the blockage of B7-H1 improved T cell-mediated immune response and tumor clearance in an ovarian cancer mouse model. VEGF exerts its effects via its receptor, VEGFR, mainly VEGFR2 [13, 131]. This type of receptor has the ability of activating the mTOR protein through the Akt/mTOR pathway [131]. Inactivation of mTOR may lead to downregulation of IL-2, thus conferring a direct negative effect in T cell proliferation as well as cancer cell proliferation [132, 133]. Except cancer cells, the VEGFR2 protein has been recently shown to be expressed selectively on a subset of T cells, namely, the CD4+ FoxP3+ Tregs [134]. Since FoxP3high Tregs are associated with poor prognosis, the expression of VEGFR2 on their surface may be attributed with a more prominent role in angiogenesis in the future. 

**Figure 3:**
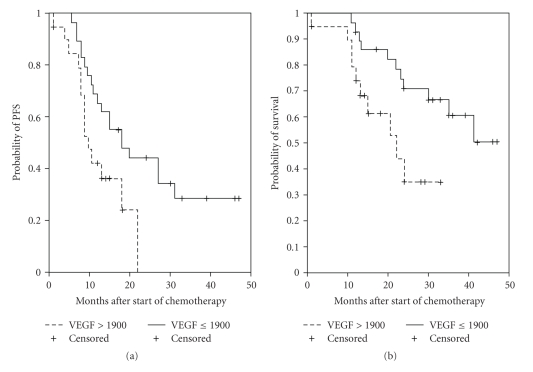
Clinical data concerning patients undertaking chemotherapy. Progression-free survival (a) and overall survival (b) of 54 patients with advanced ovarian cancer receiving first-line, platinum-based chemotherapy, according to VEGF levels in ascites. The lower levels were associated with significantly longer progression-free (*P* = .0297) and overall (*P* = .0164). Median followup: 33 months.

The prognostic significance of VEGF in ovarian cancer has received much attention recently. Several studies have associated serum or plasma levels of VEGF with prognosis [127, 135, 136]. Ascites VEGF levels may be more informative, since it reflects the site of the most intense disease activity. It has been shown that VEGF levels above 1900 pg/ml were associated with inferior survival in a series of 41 patients with advanced ovarian cancer [57, 72]. These results have been confirmed by a more recent analysis of a larger series and longer followup (Figures 3(a) and 3(b) show the updated results). Finally, in recent studies serum Fas protein (sFas) levels and serum VEGF levels have been found to be increased in ovarian cancer patients correlated with a short duration of the relapse-free period [137].

## 5. Harnessing the Immune System for Cancer Therapy: A Driven Response

In general, there are three approaches to harnessing the immune system response in order to fight cancer: (1) use exogenously administered antibodies, (2) elicit a humoral and a cellular response, and (3) explore the activation and/or generation of antigen-specific CD4+ and CD8+ cells. The strategies which are in the more advanced stages of drug development are the use of monoclonal antibodies and cytokines. The other strategies will be discussed more briefly.

Antibodies with the potential to be used in cancer treatment are often targeting either the tumor directly, the tumor microenvironment, or function as modulators of immune response [138]. Another way is to target intracellular pathway molecules by the use of cell penetrating agents [139]. Antibody immunotherapy does not seem to interfere with suppressor mechanisms that could limit its treatment capacity. Antibodies usually act by the induction of death pathways by engaging with receptors on cell surface, antibody-dependent cellular cytotoxicity (ADCC), and the blockade of tumor growth factors such as vascular endothelial growth factor (VEGF). There is a growing number of potential agents, mainly antibodies, currently undergoing evaluation in clinical trials [140]. Such antibodies include Trastuzumab [141], Oregovomab [142], Bevacizumab, and Cetuximab [143, 144]. Published data are shown in Table 1.

VEGF, mainly the VEGF-A isoform, may be the more promising therapeutic target. It is a powerful angiogenic molecule that has been associated with tumor progression, poor prognosis, and drug resistance in ovarian cancer. In addition, it has immunosuppressive properties, as previously discussed. Recent data have suggested that an anti-VEGF monoclonal antibody (Bevacizumab) is efficient in platinum-resistant disease [145–147, 154]. By combining paclitaxel and/or carboplatin agents with VEGF inhibitors, such as bevacizumab, we may overcome resistance to chemotherapy. This hypothesis is currently tested in two randomised studies [148, 149]. Both studies showed a significant PFS prolongation by the administration of Bevacizumab. Another monoclonal antibody already tested in a phase III randomized study is oregovomab, which recognizes an epitope on CA125. The formation of the oregovomab-CA125 complex results in the development of CA125-specific immune response [150]. The development of such response has been shown to predict improved survival in a small phase II study [151]. In the phase III study, although no survival advantage was found when it was given as maintenance after remission following first-line chemotherapy, subgroup analysis showed that patients with low-volume residual disease (<2 cm), Ca125 ≤ 65 IU/mL after the 3rd cycle of chemotherapy, and CA125 ≤ 35 IU/mL at entry experienced a 2-fold increase in median time to progress (TTP) [152]. The IMPACT study is currently evaluating the role of oregovomab in this subset of patients.

The use of cytokines in cancer therapy has also been evaluated. Certain cytokines, such as IFNs, can augment antitumor response and were considered as promising agents in cancer therapy. IFN-*α*, is approved for the treatment of malignant melanoma and kidney cancer. It has been shown that GM-CSF-secreting tumor cell immunotherapy with VEFG-blocking agents prolonged survival of cancer bearing mice [155, 156], while IL-2 and GM-CSF can have a suppressive effect on Tregs [92, 93]. GM-CSF in combination with recombinant IFN-*γ*1 and carboplatin in a phase II trial has been recently shown to have a reasonable response against recurrent platinum sensitive ovarian cancer [157]. All these preclinical data suggest that the use of cytokines may be efficacious in ovarian cancer. IFN is the most well-studied agent. Several randomized studies, based on promising phase II results, have been published during the last decade evaluating the role of interferon in addition to first-line therapy or as maintenance strategy. The results of these studies are summarized in Table 2. The first study showed a PFS but not OS benefit [158]. Nevertheless, the standard of Cisplatin/Cyclophosphamide, used in that study has been substituted by Paclitaxel/Carboplatin, and thus these results are difficult to be viewed in the context of current practice in ovarian cancer. Two randomized studies using the current standard showed no benefit from the addition of IFNs in the treatment of ovarian cancer [159, 160]. 

Methods to augment an immune response against tumor antigens have also been explored [161, 162]. The most studied have been vaccines or macrophage-activated killer (MAK) cells. Within this context, IFN-*γ* has recently been shown to reverse the immunosuppressive properties of macrophages so its local administration could potentially increase the efficacy of antitumor immunotherapies based on the generation of effector T cells [163], an observation that contradicts previous studies mentioned above where IFN-*γ* showed no positive effect within the tumor microenvironment. Tumor antigens, synthetic tumor peptides, whole tumor cells, tumor cell lysates, or anti-idiotypic antibodies are among the list of initiators of an immune response [161]. In some protocols, injection of synthetic peptides in combination with GM-CSF is performed. In different protocols, dendritic cells (antigen presenting cells) loaded with synthetic peptides, immunocomplexes of tumor-associated antigens with antibodies [162] through activating Fc*γ*-R [163], or fusion of dendritic cells with tumor cells are utilized. Dendritic cells present antigens to CD4+ CD8+ cells while delivering stimulatory signals necessary for effective T cell activation. They can also directly downregulate an immune response or induce immune tolerance [164]. Vaccines using either gene-modified dendritic cells or whole tumor cells have also been explored [163, 165]. Peptide vaccines have been used so far in a lesser extent since they have some important limitations [166]. Although the development of a specific immune response could be shown in patients undergoing such approaches [167, 168], their role remains investigational.

The ex vivo expansion of immunologically relevant autologous populations have also been studied. MAK cells have been used as a form of adoptive immunotherapy alone or in combination with monoclonal antibodies [58, 169, 170]. MAK can reach tumor sites by intraperitoneal infusion, but most studies are small and the role of this approach remains undetermined. Using specific CD4+ and CD8+ cells against tumor antigens may provide another way of fighting cancer. These cells need to be activated against tumor antigens before being administered to the patient. Activation can be achieved by either stimulating peripheral blood mononuclear cells (PBMC) in vitro, or by ex vivo expansion of TILs [162, 167]. Recently, the adoptive transfer of T cells expressing chimeric NKG2D receptors can lead to long-term, tumor-free survival in a murine model of ovarian cancer [171]. Genetic modification of T cells is another emerging approach but its application in ovarian cancer has not been successful so far [165]. 

Agents such as oligodeoxynucleotides containing dinucleotides with unmethylated CpG motifs (CpG-ODN) that recruit and activate innate effector cells throughout the abdominal cavity to the tumor site might control tumor cell growth and ascites formation [172]. 

Reports for the implication of Tegs in suppression of antitumor response in cancer development and prognosis have already been discussed. There are currently clinical trials using ONTAK in ovarian cancer patients, with encouraging results [15, 173]. ONTAK is a fusion toxin that consists of IL-2 genetically fused to the enzymatically active and translocating domains of diphtheria toxin. It can deplete functional Tregs, as shown by Curiel et al. [173] in ovarian cancer patients (including one patient at stage IV), by 50% in serum and it is considered to lead to better prognosis. ONTAK is approved by the FDA to be used in the treatment of CD4+ CD25+ Treg-mediated tumors.

## 6. Conclusion and Future Considerations

Both the innate and adaptive immune response can be of great importance in the battle against ovarian cancer. Throughout this paper, mechanisms of reaction of the immune system against tumors were highlighted, stressing the importance of such anti tumor response. 

The prognosis of advanced ovarian cancer has been improved in the recent years. Nevertheless, after the introduction of paclitaxel in first-line treatment, no dramatic advance in progress-free survival of the patients using cytotoxic chemotherapy can be foreseen in the immediate future. On the contrary, targeted therapies may hold a significant promise, as shown in other neoplasms. The immune response against the tumor may be a promising target, especially after much recent data has associated various elements with prognosis. 

The previous decade was characterized by many attempts to establish interferon as a standard in the treatment of ovarian cancer. The failure of those attempts stresses the disease's complexity. At the moment, monoclonal antibodies seem to be the most promising agents, currently tested in phase III trials. 

There is still much to clarify regarding the mechanisms governing the development of host antitumor response in order to find strategies to augment it. The interaction with other important functions, such as angiogenesis, may imply that more than one function needs to be blocked for achieving an efficient therapy. Further progress in basic research in combination of the awaited results of large randomized clinical trials will hopefully enrich our armamentarium against ovarian cancer. 

##  Conflict of Interests

No conflict of interests is to be reported.

## Figures and Tables

**Table 1:** Selected clinical studies of monoclonal antibodies used for the treatment of ovarian cancer.

Antibody	Mechanism of action	Representative Phase II studies			Phase III studies		
		Population	Treatment	Results	Population	Treatment	Results
Bevacizumab (Genentech/Roche)	Binds to VEGF Antiangiogenic Immunosuppressive	Refractory (*n* = 32) [145]	Monotherapy (*n* = 23) With chemotherapy (*n* = 9)	RR 16% PFS 5.5 m OS 6.9 m	First-line ICON 7 [146]	Carboplatin/Paclitaxel versus Carboplatin/Paclitaxel/Bevacizum ab	Median PFS 17.3 m versus 19 m, *P* = .0041 10.3 m versus 11.2 m versus 14.1, *P* < .00001
		Refractory (*n* = 44) [147]	Monotherapy	RR 16% PFS 4.4 m OS 10.7	GOG 218 [148]	Carboplatin/Paclitaxel versus Carboplatin/Paclitaxel/Bevacizum ab versus Carboplatin/Paclitaxel/Bevacizum ab + Bevacizumab maintenance	

Oregovomab (AltaRex Corp)	Binds to CA125 Development of a humoral and cellular antitumor response	2nd line treatment (*n* = 20) [149]	With chemotherapy	Development of T cell response was associated with improved survival	Maintenance after first-line (*n* = 147) [150]	Oregovomab versus placebo	Median PFS 13.3 m versus 10.3 m, *P* = .71

					Maintenance after first-line Residual<2 cm, CA125 < 65 after 3rd cycle, CA125 < 35 at entry (*n* = 354)	Oregovomab versus placebo	Awaited

Trastuzumab (Genentech)	Binds to HER2 extracellular domain	Recurrent (*n* = 41) [141]	Monotherapy	RR 7.3% PFS 2 m			

Pertuzumab (Genentech)	Inhibitor of HER dimerization	87% platinum-resistant (*n* = 123) [151]	monotherapy	RR 4.3% PFS 6.6 w			

Cetuximab (Bristol-Myers Squibb)	EGFR inhibitor	First-line (*n* = 41) [152]	Combination with paclitaxel/carboplatin	PFS 14.4 m			

Matuzumab (Merck/Serono/Takeda)	EGFR inhibitor	Platinum-resistant (*n* = 37) [153]	Monotherapy	RR 16.2 m TTP 54d OS 13.3 m			

**Table 2:** Selected clinical studies of cytokines for the treatment of ovarian cancer.

Cytokine	Phase III studies		
	Population	Treatment	Results
IFN-*γ*	First line (*n* = 148) [156]	Cisplatin/Cyclophosphamide versus Cisplatin/Cyclophosphamide/IFN*γ*	3-year OS 58% versus 74% (*P* = .23) 3-year PFS 38% versus 51% (*P* = .031)

IFNa-2a	Maintenance after first-line (*n* = 300) [157]	IFNa-2a versus Observation	No benefit

IFN-*γ*	First line (*n* = 847) [158]	Carboplatin/Paclitaxel versus Carboplatin/Paclitaxel/IFN-*γ*	Median OS Not estimated versus 1138d HR: 1.45, *P* = .001
